# The role of IL‐6/JAK/STAT signal in female infertility caused by hydrosalpinx

**DOI:** 10.1002/iid3.871

**Published:** 2023-06-28

**Authors:** Jia Deng, Yufu Huang, Luying Wang, Xin Sun

**Affiliations:** ^1^ Department of Gynecologic The Third Xiangya Hospital of Central South University Changsha Hunan Province China

**Keywords:** IL‐6, JAK/STAT signaling pathway, pelvic inflammatory disease, STAT3, tubal infertility

## Abstract

**Introduction:**

To explore the role of IL‐6/JAK/STAT signaling in tubal infertility.

**Methods:**

The fimbriae tissues of 14 patients with a history of infertility and hydrosalpinx and 14 patients with no history of infertility and no fallopian tube disease were collected. The tissues were then divided into hydrosalpinx group and control group followed by analysis of the protein expression of key factors in the IL‐6/JAK/STAT signaling by immunohistochemistry and Western blot.

**Results:**

Immunohistochemical staining showed significantly higher level of IL‐6, JAK1, p‐JAK1, JAK2, p‐JAK2, STAT1, p‐STAT1, STAT3, and p‐STAT3 in hydrosalpinx group than those in control group with IL‐6 being mainly located in the cytoplasm and p‐JAK2, STAT1, p‐STAT1, STAT3, and p‐STAT3 in the cytoplasm and nucleus. JAK1 and p‐JAK1 was mainly located in the cytoplasm and JAK2 is in the cytoplasm and nucleus without difference of their expression between two groups. Consistently, hydrosalpinx group presented significantly higher protein levels of IL‐6, JAK1, p‐JAK1, JAK2, p‐JAK2, STAT1, p‐STAT1, STAT3, and p‐STAT3 than control group without difference of JAK1, p‐JAK1, JAK2 level.

**Conclusion:**

The activation of IL‐6/JAK2/STAT1 and STAT3 signaling pathways are found in the hydrosalpinx in infertile patients, indicating that they might be involved in the pathogenesis of hydrosalpinx.

## Graphical abstract

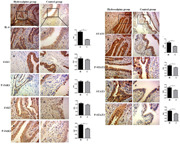

## 1 INTRODUCTION

Tubal infertility is one of the main causes of female infertility. Infertility caused by congenital structural development of the fallopian tube or acquired factors that cause inflammation, adhesion, and obstruction of the fallopian tube is called tubal infertility.^28^ According to statistics, in developing countries, one out of every four couples have infertility, of which female factors account for a half, while tubal infertility accounts for about 10%–30% of female infertility.^1^ Hydrosalpinx is the most serious damage, accounting for 15%–20% of patients with tubal infertility.^2^ At present, drug treatment is difficult in clinic and there are several surgical treatment methods, but the outcomes are not satisfactory. Multiple factors have been identified to contribute to tubal infertility, among which pelvic inflammatory disease (PID) is the main factor, and the occurrence of PID is mostly related to sexually transmitted diseases, such as: Chlamydia trachomatis, Neisseria gonorrhoeae, Mycoplasma, and so forth. Usually these pathogens (such as chlamydia) can infect female lower genital tract epithelial cells through sexual transmission, leading to cervicitis, and then through the cervix to infect the upper genital tract, induce upper genital tract tissue damage, and cause persistent infection, causing secondary inflammatory diseases of the pelvic cavity,^3^ especially causing persistent inflammation of the fallopian tube, causing congestion and edema of the epithelial cells of the fallopian tube, impaired ciliary motility, blockage of the fallopian tube tissue, adhesion of the surrounding tissue, and atresia of the umbrella end causing hydrosalpinx, and finally lead to infertility.^4^

Interleukin 6 (IL‐6) is a cytokine with multifunctional activities. It is produced by monocytes, macrophages, lymphocytes, fibroblasts, keratinocytes, endothelial cells, and certain tumor cells. It participates in the body's inflammatory response and immunity as well as in cell proliferation and apoptosis.^29^ Current studies have found that Interleukin 6R (IL‐6R) has two forms, one is the transmembrane receptor protein mIL‐6R and the other is the soluble receptor protein sIL‐6R.^30^, ^31^ After IL‐6 binds to receptor, a complex IL‐6/IL‐6Ra is formed, which activates another transmembrane glycoprotein, G protein coupled receptor (gp‐130). IL‐6 binds to the receptor IL‐6Ra in two ways, the classic or the cis‐signaling pathway and the trans‐signaling pathway. These two ways of binding play distinct biological functions in cells. The cis‐transduction pathway is considered to be involved in the anti‐inflammatory response, while the trans‐transmission pathway is considered to be involved in the pro‐inflammatory response and tumor formation, so trans‐signaling pathway is often a drug target for controlling inflammation and treating tumors. At present, studies have found that IL‐6 is significantly increased in the serum and peritoneal effusion of patients with tubal infertility,^8^, ^9^ and Chlamydia trachomatis was used to infect mice to cause fallopian tubes water and IL‐6 was found to be related to the pathogenicity of chlamydia during this process.^10^ IL‐6 involves in signal transduction in cells through multiple signal pathways in the body. Janus kinase (JAK)/signal transducer and activator of transcription (STAT) signal pathway is one of its main signal pathways. Current studies have found that IL‐6 mediates JAK/STAT signal pathway and participates in multiple diseases, including inflammatory diseases, autoimmune diseases, cancer.^11^, ^12^, ^13^, ^14^, ^15^ In recent years, clinical specific inhibitors for related factors of this pathway have been developed to treat diseases. Therefore, we speculate that the inflammatory immune response mediated by IL‐6/JAK/STAT signaling pathway may be involved in the formation of hydrosalpinx and lead to tubal infertility. In this study, immunohistochemistry and Western blot methods were used to detect the protein expression of IL‐6/JAK/STAT signaling proteins in human fallopian tube fimbria tissues to explore the role of this signaling pathway in tubal infertility.

## 2 MATERIALS AND METHODS

### 2.1 Patients

From November 2018 to September 2019 in our hospital, at least one hydrosalpinx tube fimbria part of the tissue in 14 cases confirmed by laparoscopic surgery were collected hydrosalpinx group. In the same period, 14 cases of the tissue of the fimbriae of the fallopian tube were selected as the control group.

The inclusion criteria for patients in hydrosalpinx group are: (1) have regular sex without contraception and have not been pregnant for 1 year or more; (2) B‐ultrasound or hysterosalpingography indicates hydrosalpinx; (3) Laparoscopy confirms that there is at least one hydrosalpinx, and IVF is planned‐ ET requires bilateral fallopian tube ligation; (4) Liver and kidney function, inflammatory indicators (white blood cell, neutrophil percentage, etc.) are all normal. Inclusion criteria for patients in the control group: (1) No history of infertility; (2) Laparoscopic surgery is required for other benign noninflammatory diseases, and at least one fallopian tube is required to be removed during the operation; (3) No hydrosalpinx and chronic hydrosalpinx are confirmed under laparoscopic surgery Inflammatory lesions; (4) The liver and kidney function, inflammatory indicators (white blood cell, neutrophil percentage, etc.) are normal. Common exclusion criteria: systemic infectious diseases or malignant tumor diseases; acute infection period; menopausal women; endometriosis; autoimmune diseases.

Hydrosalpinx group/control group specimens: the fimbriae tissue of the fallopian tube was washed with sterilized water and divided into two, one was stored at −80°C for total protein extraction, and the other was directly fixed in 4% paraformaldehyde to prepare paraffin tissue sections.

### 2.2 Immunohistochemistry

The fimbriae tissue fixed in 4% paraformaldehyde solution was taken out, dehydrated, transparent, embedded in wax, and trimmed in accordance with the conventional alcohol gradient followed by continuous sectioning at a thickness of 4 μm, removal of the wax slices. After prebaking, dewaxing, hydration, antigen retrieval, and sealing, the slice was incubated with primary and secondary antibodies (39.20°F) followed by addition of 50 μL streptomyces antibiotics‐peroxidase solution, washing, drying the slides. After that, DAB color development solution was added and the color development time was adjusted under the microscope, rinse the sections with tap water to stop the color development. After counterstaining with hematoxylin for 5 min, rinse with tap water again and then return to blue with PBS solution. Put the slices ascending steps into ethanol solution for dehydration and air drying followed by mounting the slices.

Under a microscope, the overall tissue morphology of the slice and the expression of positive factors was observed under a microscope (×100), and then randomly selected a field of view (×400) to save images. Image‐pro plus image analysis software calculated the integrated optical density (IOD) of the positive part and the average optical density value (mean density), namely IOD/Area.

### 2.3 Western blot

The tissue block was washed twice with precooled PBS, and about 0.5 mg of tissue was cut with scissors, placed in a tissue homogenizer, and 1 mL of total protein extract was added to extract the protein which was quantified by BCA kit and separated on the SDS‐PAGE for western blot using relevant antibodies, incubated overnight at 39.20°F. The membrane was developed and exposed with ECL reagent.

### 2.4 Statistical methods

SPSS 20.0 software analyzed data which were shown as mean ± standard deviation and assessed by independent sample *t* test or a nonparametric test (Mann–Whitney *U* test). *p* < .05 indicates a difference.

## 3 RESULTS

### 3.1 Immunohistochemical analysis of IL‐6/JAK/STAT signaling proteins

**Figure 1:**
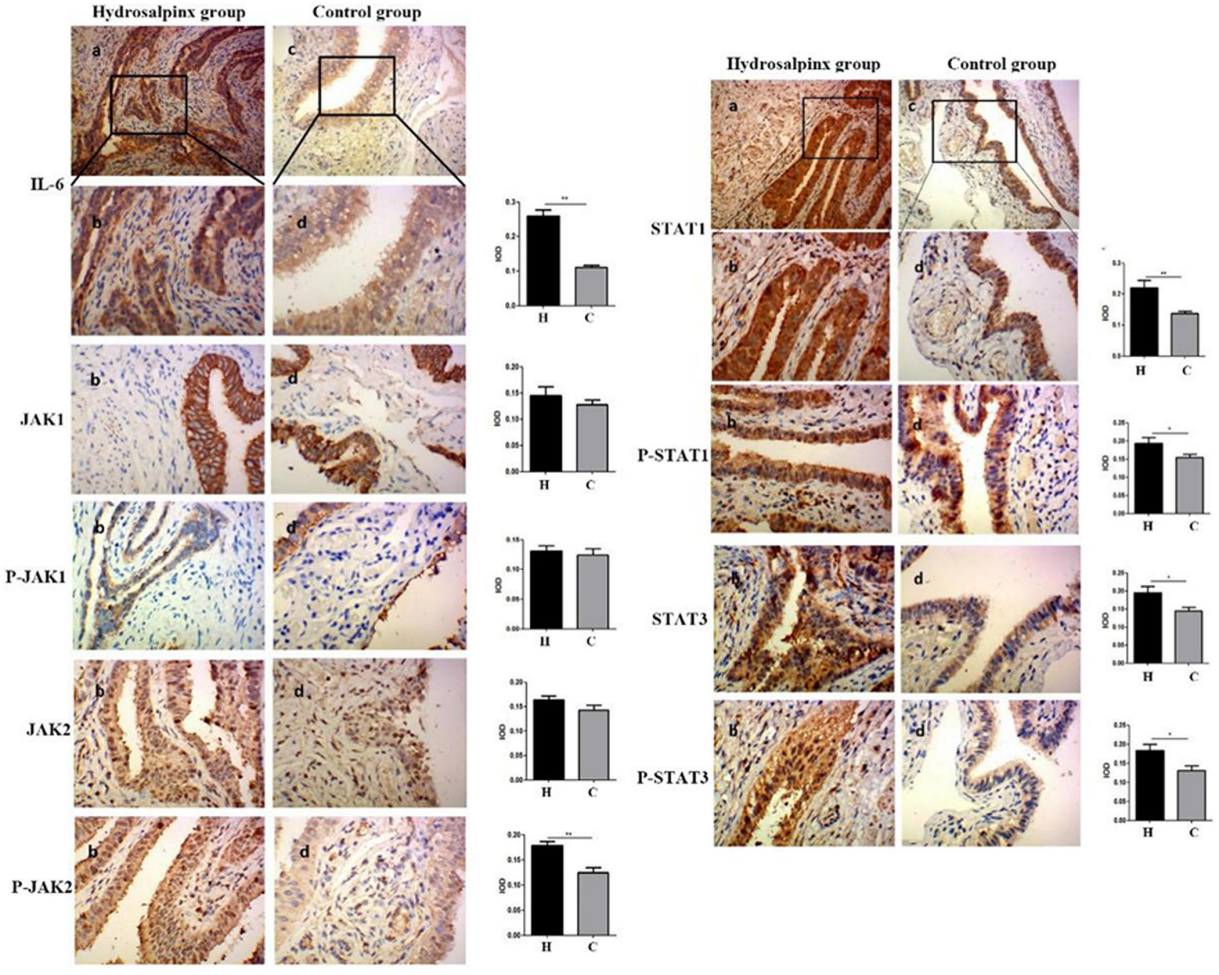
The expression of key factors of IL‐6/JAK/STAT signaling pathway in the hydrosalpinx group and control group. Hydrosalpinx group (H): (A) (×100) and (B) (×400); Control group (C): (C) (×100), and (D) (×400). Positive staining was shown as brownish yellow, dark to brown, *p < .05, **p < .01.

Through immunohistochemical detection, IL‐6, JAK1, p‐JAK1 was mainly located in the cytoplasm, and JAK2, p‐JAK2, STAT1, p‐STAT1, STAT3, and p‐STAT3 were mainly located in the cytoplasm and nucleus (Figure 1). Quantification of IOD/Area showed significantly higher levels of above factors in hydrosalpinx group than control group (*p* < .05). However, the expression of JAK1, p‐JAK1, and JAK2 in the hydrosalpinx group showed no differences from that in control group (*p* > .05) (Table 1).

**Table 1:** Related factors expression of IL‐6/JAK/STAT signal pathway (IOD/Area).

	Hydrosalpinx group (*n* = 14)	Control group (*n* = 14)	t/Z	*p* Value
IL‐6	0.258 ± 0.069	0.111 ± 0.018	7.742	<.001*
JAK1	0.145 ± 0.062	0.127 ± 0.036	0.905	.374
P‐JAK1	0.131 ± 0.033	0.125 ± 0.038	0.473	.640
JAK2	0.164 ± 0.031	0.143 ± 0.036	1.637	.114
P‐JAK2	0.179 ± 0.031	0.115 (0.098,0.134)	−3.308	.001*
STAT1	0.220 ± 0.086	0.137 ± 0.026	3.453	.003*
P‐STAT1	0.193 ± 0.060	0.155 ± 0.030	2.155	.044*
STAT3	0.194 ± 0.066	0.144 ± 0.038	2.460	.021*
P‐STAT3	0.184 ± 0.058	0.131 ± 0.044	2.697	.012*

*Note*: The P‐JAK2 data of the control group did not conform to the normal distribution, and the data were represented by the median and interquartile range. The Mann–Whitney *U* test was used for comparison between two groups. The cofactors were all conformed to the normal distribution, and the data were shown as the mean ± standard deviation and assessed by independent‐sample *t* test.

Abbreviation: IOD, integrated optical density.

* The difference is statistically significant.

### 3.2 Western blot detection of IL‐6/JAK/STAT signaling proteins

**Figure 2:**
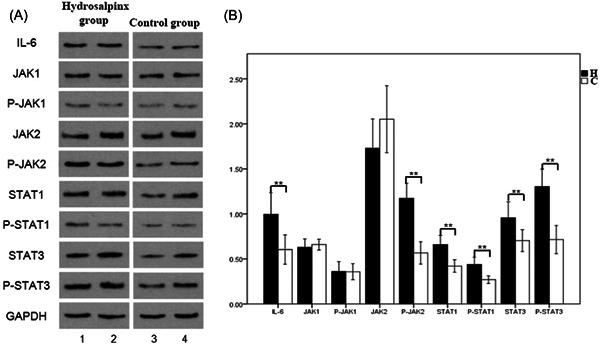
Protein expression of related factors in IL‐6/JAK/STAT signaling pathway. (A) Representative western blot images. 1 and 2: Hydrosalpinx group (H). 3 and 4: Control group (C). (B) Semiquantitative analysis. *p < .05, **p < .01.

Western blot analysis showed the positive expression of IL‐6, JAK1, p‐JAK1, JAK2, p‐JAK2, STAT1, p‐STAT1, STAT3, and p‐STAT3 in hydrosalpinx group and control group (Figure 2). By calculating the gray value of the target protein relative to GAPDH, it was found that IL‐6, p‐JAK2, STAT1, p‐STAT1, STAT3, and p‐STAT3 levels were significantly higher in hydrosalpinx group than control group (*p* < .05). However, JAK1, P‐JAK1, and JAK2 showed no differences between these two groups (*p* > .05) (Table 2). In addition, hydrosalpinx group showed significantly higher total STAT3 than total STAT1, as well as higher p‐STAT3 than p‐STAT1 (*p* < .05) (Table 3).

**Table 2:** IL‐6/JAK/STAT signal proteins expression (target protein/GAPDH).

	Hydrosalpinx group(*n* = 14)	Control group(*n* = 14)	t/Z	*p* Value
IL‐6	1.070 ± 0.329	0.689 ± 0.257	3.407	.002*
JAK1	0.666 ± 0.144	0.659 ± 0.103	0.136	.893
P‐JAK1	0.396 ± 0.171	0.359 ± 0.154	0.603	.552
JAK2	1.970 (1.720,2.090)	2.052 ± 0.644	–0.919	.358
P‐JAK2	1.256 ± 0.267	0.567 ± 0.214	7.524	<.001*
STAT1	0.605 (0.560,0.913)	0.422 ± 0.121	–3.473	.001*
P‐STAT1	0.504 ± 0.173	0.270 ± 0.073	4.649	<.001*
STAT3	1.059 ± 0.285	0.704 ± 0.209	3.744	.001*
P‐STAT3	1.315 ± 0.322	0.715 ± 0.271	5.336	<.001*

*Note*: The JAK2 and STAT1 data of the hydrosalpinx group did not conform to the normal distribution, and the data were represented by the median and interquartile range. The comparison between the two groups was performed by the Mann–Whitney *U* test. The cofactors were all conformed to the normal distribution, and the data were shown as mean ± standard deviation and assessed by independent sample *t* test.

* The difference is statistically significant.

**Table 3:** STAT1 and STAT3 protein expression in hydrosalpinx group.

	STAT1 (*n* = 14)	STAT3 (*n* = 14)	t/Z	*p* Value
Total protein level	0.605 (0.560,0.913)	1.059 ± 0.285	–3.127	.002*
Phosphorylation level	0.504 ± 0.173	1.315 ± 0.322	–8.308	<.001*

*Note*: The total STAT1 data did not conform to the normal distribution, and the data were expressed in median and interquartile range. The comparison between the two groups was conducted by the Mann–Whitney *U* test. The cofactors were in conformity with the normal distribution, and the data were expressed in the mean ± standard deviation. The relative expression of phosphorylated protein was compared between the two groups using independent sample *t* test.

* The difference is statistically significant.

## 4 DISCUSSION

Tubal infertility is one of the main causes of female infertility. After ovulation, the ovary needs to be picked up by the fimbriae of the fallopian tube to enter the fallopian tube and bind with the sperm. The transportation process of the egg and sperm mainly depends on the activity of the cilia of the fallopian tube epithelial cells and the contraction of the smooth muscle of the fallopian tube. Any factors that affect the above process may cause tubal infertility.^16^ Pelvic inflammatory disease is one of the main reasons, so the inflammatory immune response mediated by inflammatory factors is the focus of our research. IL‐6 can not only play an anti‐inflammatory effect but also promote inflammatory response through binding with receptors. In this study, IL‐6, p‐JAK2, STAT1, STAT3, p‐STAT1, p‐STAT3 level in the mucosal layer cells of the fimbriae tissue of the fallopian tube in the hydrops group was significantly higher than that in the control group, indicating that IL‐6 may signal through the JAK2/STAT1 and STAT3 pathways to promote the chronic inflammation of the fallopian tube and cause the fallopian tube Stagnant water.

Studies have found that the fallopian tube itself contains abundant macrophages and dendritic cells. When pathogens are infected through the lower reproductive tract, the pattern receptors on the cell surface will first recognize harmful stimuli. At this time, dendritic cells can effectively function as antigen presentation and first‐line defense, and it will cause acute inflammation and innate immune response of the body, recruit innate immune cells, such as neutrophils, macrophages, and so forth, and secrete complement and cytokines to participate in the immune response.^5^, ^17^ If the pathogen persists and is not cleared by the body, it will cause chronic pelvic infection and activate adaptive immunity, during which IL‐6 will continue to be secreted. In the acute inflammatory response, the infiltration of neutrophils plays a major role, and after that, it is transformed into monocytes predominantly. This also indicates that acute inflammation is transforming into chronic inflammation, and in this transition process, the trans conduction pathway mediated by the IL‐6/sIL‐6R complex plays an important role. IL‐6 activates endothelial cells to secrete monocyte chemotactic protein (myxovirus resistance 1, MCP‐1), which leads to the recruitment of monocytes and causes specific immune responses, promotes the differentiation of B cells and activation of T cells. Acute inflammation is the body's defense mechanism against harmful external stimuli, while chronic inflammation can cause damage to surrounding tissues.^17^, ^18^ In the past, studies have found that IL‐6 is increased in the serum and abdominal effusion of patients with tubal infertility,^8^, ^19^ and high concentrations of IL‐6 can inhibit the activity of tubal epithelial cilia.^20^ This study found that IL‐6 relative expression was significantly higher than that in the control group, proving that IL‐6 might be involved in tubal infertility.

The JAK/STAT signal pathway is one of the main pathways of IL‐6 downstream signal transduction. Our detection of the key factors of this pathway found that the total protein of JAK2 has no significant difference between the two groups, while the expression of P‐JAK2 in the stagnant water group However, it is significantly higher, which indicates that IL‐6 mainly activates downstream signaling pathways in this process by affecting the phosphorylation of JAK2. JAK protein contains seven Janus homology domains. From the structural analysis, the JH1 domain of JAK2 is a highly conserved kinase domain that can regulate the activity and function of JAK2. The JH2 domain is a pseudokinase domain with a low catalytic activity and can negatively regulate the JH1 domain. In addition, under normal circumstances, JH1 is mainly responsible for the phosphorylation of substrate proteins, while JH2 can auto‐phosphorylate JAK2 when there is no stimulation by other cytokines, so that JAK is maintained in a low activity state.^21^, ^22^ In this study, it was found no significant difference JAK2 expression between hydrosalpinx group and control group, but the level of p‐JAK2 was significantly increased. Through the structural analysis of JAK2, the P‐JAK2 in control group maintained its physiology due to the action of the JH2 domain. Under the condition of low phosphorylation, and the excessive stimulation of IL‐6 in the hydrosalpinx group promoted the phosphorylation of JAK2 and caused the conduction of JAK/STAT signaling pathway, but in this process, the total protein level of JAK2 was not affected.

Current studies have found that STATs produced by different cytokines have different effects on the body, and STAT3 produced under IL‐6 stimulation does play a major role in promoting inflammation.^23^ The IL‐6‐mediated JAK‐STAT signaling pathway mainly activates STAT1 and STAT3, among which STAT3 has a higher affinity than STAT1, and in the absence of STAT1, IL‐6 cytokine stimulation did not increase STAT3 expression, which indicates that STAT1 has no effect on the expression of STAT3 under IL‐6 stimulation.^24^ In inflammation and tumors, IL‐6‐mediated STAT3 can also participate in the autonomous migration of cells by regulating growth factors, forming an inflammation cascade amplification effect and tumor metastasis.^25^ In this study, the levels of STAT3 and p‐STAT3 in hydrosalpinx group were significantly higher than those in control group, suggesting that STAT3 produced by excessive stimulation of IL‐6 cytokines plays an important role in causing inflammation of the fallopian tubes. As mentioned above, the fallopian tube is rich in macrophages. When the body is stimulated, the macrophages in the resting state can be polarized. They are divided into two types: M1 and M2. The M1 type mainly plays a role in promoting inflammation and can secrete a large amount of pro‐inflammatory factors, release a large amount of NO and ROS, thereby killing pathogens. M2 mainly plays a role in inhibiting the inflammatory response, secreting IL‐10 and other factors to inhibit the response, thereby promoting tissue repair. The balance of M1 and M2 plays an important role in the inflammatory response.^26^ During pathogens infection, it can stimulate macrophages to secrete a large amount of pro‐inflammatory factors including IL‐6, and IL‐6 stimulates cells to express a large amount of STAT3 which can promote the polarization of macrophages to M1 type, and further promote the release of a large number of inflammatory factors. This positive feedback effect further promotes the polarization of M1 macrophages, so that in the later stage of acute inflammation, M0 type macrophages cannot transform to M2 type, breaking balance between M1 and M2 type macrophages, resulting in persistent chronic inflammation of the fallopian tube leading to tissue damage.^5^, ^27^ In addition, STAT3 can stimulate the differentiation of naive T cells into Th17 cells in the inflammatory response, thereby producing IL‐17 to promote the inflammatory response.^6^

STAT1 is mainly activated by IFNγ and IFNα in the body. The activation rate of STAT1 by IL‐6 is much lower than that of IL‐6. Under the stimulation of IL‐6, it mainly produces STAT1/STAT1, STAT3/STAT3, and STAT1/STAT3.^24^ In this study, it was found that the content of STAT3 was significantly higher than that of STAT1, which is similar to the results of previous studies, but previous studies also found that STAT1 can only be phosphorylated temporarily under the stimulation of IL‐6 alone, while STAT3 can be continuously phosphorylated. For patients with hydrosalpinx, this is a process of chronic inflammation that persists and may be related to the p‐STAT1 homodimer entering the nucleus, which can interact with IFN‐stimulated genes promoters to induce the expression of IFNγ. Although p‐STAT1 can only play a short‐term role, the continuous activation in this process produces U‐STAT1, which is the key to subsequent effects. U‐STAT1 is expressed horizontally and can continuously induce the production of OAS, RNASEL, PKR, MX1, and other proteins to promote inflammation.^7^, ^24^ Phosphorylated STAT1 homodimers need to rely on the interaction of α, β import proteins and nucleoporins and consume a certain amount of energy to complete, while U‐STAT1 can directly enter the nucleus through nucleoporins, which is more conducive to promoting inflammation. In this study, it was found that STAT1 and p‐STAT1 in the hydrosalpinx group were higher than those in the control group, which proved that STAT1 involves in the hydrosalpinx.

In conclusion, JAK/STAT signal pathway mediated by IL‐6 involves in hydrosalpinx, indicating that they might be novel targets for the treatment of hydrosalpinx.

## AUTHOR CONTRIBUTIONS

Xin Sun was dedicated to the integrity of the entire study, study concepts, study design, clinical studies, manuscript editing and manuscript review. Jia Deng focused on the study design, literature research, experimental studies, data acquisition, data analysis, statistical analysis, manuscript preparation. Yufu Huang was involved in the experimental studies. Luying Wang carried out the clinical studies. All authors have read and approved this article.

## CONFLICT OF INTEREST STATEMENT

The authors declare no conflict of interest.

## ETHICS STATEMENT

This study is approved by the Ethics Committee of Third XiangYa Hospital, Central South University (No: 2017‐S168). Informed consent was obtained from all individuals included in this study.

## Data Availability

All data generated or analysed during this study are included in this. Further enquiries can be directed to the corresponding author.
